# On Dyspepsia of Liquids

**Published:** 1872-04

**Authors:** 


*On Dyspepsia of Liquids.*

Dr. Thorowgood records (*Lancet*, Feb. 17, 1872) several cases which he considers to be good illustrations of that indigestion of liquids which has been carefully described by Chomel in his work on Dyspepsia. The affection, in its fully developed form, he does not believe is common in this country, though he has met with several cases in the course of the last few years. The following is one of them:

James M'C., aged 30, a pale, dark, intelligent man, came under Dr. Thorowgood's care, complaining generally of dyspeptic symptoms, and especially of the great uneasiness caused by the presence of any amount of liquid in the stomach. Liquids in the slightest degree acid were most distressing to him, and at times he had attacks of sour pyrosis. He complained much of dryness of mouth, with dry skin and costive bowels, urine loaded with lithates, but free from traces alike of sugar or albumen. No loss of flesh; pulse slow and soft; nothing irregular to be found in heart or lungs; no sort of tumor or thickening about pylorus, but on gently vibrating the stomach, fluid was heard splashing about in it, and this sound could be always produced irrespectively of any liquid having been recently ingested. The stomach was much distended. The early treatment of this case consisted in the use of alkalies with bismuth and various bitters, but no improvement resulted; the only noteworthy feature was the effect of a pill of extract of opium at night, which regulated the action of the bowels so completely that the patient asked for the pills as *aperient* pills. It was afterwards agreed that the patient should drink as little as possible, and take no other medicine than a powder of rhubarb and magnesia every morning. From this time he steadily improved, and after about two months' treatment he appeared to be cured, and was, six months afterwards, still in good health.

Dr. Thorowgood considers the dry plan of diet the only one likely to be followed by any amelioration of the symptoms; and until this is tried, no medicine will prove of any service. At times fits of faintness, with irregular action of the heart, are prominent symptoms in these cases of dyspepsia of liquids, the cause being due to the distended state of the stomach. The patient must bear a certain amount of thirst as well as he can, and take but small quantities of liquids at a time, and not drink for an hour or more after he has taken his meal of solid food. Weak whisky and water, sherry wine, and toast and water, are amongst the least objectionable drinks; and sometimes a small cup of good beef-tea, free from any farinaceous admixture, will suit well. One of his patients found a wineglass of good stout to agree well and relieve his thirst.—*Practitioner*, March, 1872.

